# Language Impairment From 4 to 12 Years: Prediction and Etiology

**DOI:** 10.1044/2013_JSLHR-L-12-0240

**Published:** 2014-06-01

**Authors:** Marianna E. Hayiou-Thomas, Philip S. Dale, Robert Plomin

**Affiliations:** a University of York, United Kingdom; b University of New Mexico, Albuquerque; c King’s College London, United Kingdom

**Keywords:** language impairment, etiology, genetics, longitudinal, prediction

## Abstract

**Purpose:**

The authors of this article examined the etiology of developmental language impairment (LI) at 4 and 12 years of age, as well as the relationship between the 2.

**Method:**

Phenotypic and quantitative genetic analyses using longitudinal data from the Twins Early Development Study (Oliver & Plomin, 2007) were conducted. A total of 2,923 pairs of twins (1,075 monozygotic [MZ]; 975 dizygotic same sex [DZss]; and 873 dizygotic opposite sex [DZos]) provided data at 4 and 12 years. At 4 years, (a) psychometric LI was defined on the basis of a low parent-reported expressive vocabulary score (−1.25 *SD*s; 226 MZ and 115 DZss probands for genetic analysis); and (b) parent referral was defined as having seen a medical professional or speech-language pathologist following parental concern (112 MZ and 104 DZss probands). The 12-year language measure was a composite of 4 web-administered receptive language tests.

**Results:**

(a) Psychometric LI at 4 years is more predictive than parent referral of poor language performance at age 12 years, and (b) parent referral is substantially and significantly more heritable than psychometric LI.

**Conclusions:**

Parents’ concern about their child’s language development seems to be the marker of a more heritable disorder than poor expressive language skills alone. However, the language difficulties that arouse parental concern in preschool children, although more heritable, are not predictive of language difficulties in early adolescence. Rather, poor expressive language skills at age 4 years, psychometrically defined, are a better predictor than parent referral of continuing language difficulties at age 12 years.

The pace of early language acquisition is highly variable: The age at which the majority of children (10th–90th percentile) say their first word varies from 10 to 16 months, with a substantial “late” tail out to 24 months and beyond (Fenson et al., 1994). There are similarly wide individual differences in the speed with which children acquire a functional vocabulary and begin to comprehend and produce complex utterances (Fenson et al., 1994). Children who are slow to begin talking may arouse their parents’ concern, and late talkers form a substantial proportion of referrals to clinical services for toddlers. Children who are reported to produce fewer than 50 words and/or no word combinations at 24 months are typically considered late talkers. However, many of these children make up for their slow start, and their apparent early language difficulties seem to spontaneously resolve over the next 2 years or so (Dale, Price, Bishop, & Plomin, 2003; Rescorla, 2002; Rescorla & Dale, 2013), whereas others will have continuing deficits that may lead to a later diagnosis of language impairment (LI), whether specific or more general.

This variable pattern of spontaneous recovery means that it is very difficult to make predictions about the likelihood of future language difficulties on the basis of 2-year-old language skills (Dale et al., 2003). After the age of 4 years, however, it is generally assumed that there will be more stability and that language difficulties at this age are more likely to persist. In the current article, we use a genetically sensitive design to examine the issue of the stability of LI after the age of 4 years. We consider how well LI at 4 years of age can predict LI at 12 years, and whether using different diagnostic criteria for LI at 4 years affects the strength of this prediction. We compare the etiology of LI at 4 and 12 years in terms of the relative contributions of genetic and environmental influences; and further break down these categories of LI to examine transient, persistent, and late-onset LI. Finally, we examine whether the same genetic and environmental factors underlie LI at these two very different ages.

## Persistence of Early Language Delay and the Importance of Diagnostic Criteria

Two closely related issues of particular relevance to the current study are (a) the persistence of early LI and (b) the diagnostic criteria that are used to identify LI. Relatively few long-term studies of LI, that is, extending over more than 2 or 3 years, have been conducted, and most of them have been based on following clinically defined samples. Only recently have there been studies of population-based samples, which have at least two important advantages. First, these studies are more likely to include children with mild-to-moderate impairments, who may not have been clinically referred. Second, these studies permit the study of predictors of outcome, LI or otherwise, for children who did not manifest impairment at the early age.

It is difficult to draw firm conclusions about the outcomes of early LI from the existing literature because studies have varied widely in the criteria used for the initial judgment of LI, including (a) the areas of language affected (e.g., receptive vs. expressive), (b) the strictness of the criterion (e.g., −1 *SD* vs. lowest 10%), (c) whether the later classification used a similar or different measure of language, (d) the measurement error of the language instruments, and (e) demographic and other cohort characteristics. Thus, in looking at longitudinal studies extending for at least 4 years, the estimates of persistence of early LI range from 39% (Silva, McGee, & Williams, 1983) to 75% (Botting, Faragher, Simkin, Knox, & Conti-Ramsden, 2001). Nevertheless, there is good agreement that early LI is a substantial risk factor for later LI, even though there is in every case considerable variability in outcome, the predictors of which are poorly understood.

Several generalizations about LI do emerge, in at least a tentative sense, from current research. One is that the broader the impairment (e.g., both receptive and expressive vs. just one of them, or adding a nonverbal impairment), the more likely it is that the LI will persist (Bishop & Edmundson, 1987; Beitchman, Wilson, Brownlie, & Walters, 1996; Tomblin, Zhang, Buckwalter, & O’Brien, 2003). Another is that population-based samples are likely to show lower rates of persistence (Silva et al., 1983; the Twins Early Development Study (TEDS; Oliver & Plomin, 2007), perhaps reflecting the greater inclusion of mild impairments at the early age. A third is that children with early LI are likely to have weaker skills at the later age even if they do not qualify as having an LI, especially in certain areas such as phonological awareness and reading (Rescorla, 2005; Stothard, Snowling, Bishop, Chipchase, & Kaplan, 1998; Tomblin et al., 2003). Finally, there is clear evidence for a variety of development trajectories for children with LI: Some show a stable pattern of slower growth; some show at least a temporary spurt and catch up, though this may be followed by a plateau; and some show a lower intercept but similar slope to typically developing children (Beitchman et al., 1996; Bishop & Edmundson, 1987; Law, Tomblin, & Zhang, 2008; Stothard et al., 1998).

It should also be recognized that estimated rates of LI persistence may underestimate the true rate, as a substantial amount of apparent “recovery” may actually be regression to the mean, as Tomblin et al. (2003) argued. By using a separate baseline measure of language independent of the measure used for diagnosis, Tomblin et al. was able to show that the majority of change over the 4 years of the study relative to the initial diagnostic measure was in fact due to regression to the mean. In contrast, there was very little change relative to the independent baseline measure, suggesting strong persistence.

## Heritability of Language Skills and LI: Previous Work

Given the high degree of variability in outcome with respect to both the absolute level and pattern of development, etiological research, which has the potential to distinguish genetic and environmental influences, holds considerable promise. Recent work from large-scale studies, primarily TEDS and the International Longitudinal Twin Study (ILTS; Samuelsson et al., 2005), suggests that individual differences in language skills in young preschool children are subject to both genetic and shared environmental influences but that these vary for different components of the language system. In broad terms, variation in vocabulary and grammatical skills seems to be largely attributable to shared environmental factors (accounting for approximately two thirds of the variance), with a significant but more modest contribution from genetic factors (approximately one quarter of the variance). By contrast, phonological skills seem to be influenced to a greater extent by genetic factors, with shared environmental factors playing a lesser role (Byrne et al., 2006; Hayiou-Thomas et al., 2006; Samuelsson et al., 2005; Spinath, Price, Dale, & Plomin, 2004).

Fewer studies have examined the heritability of language skills in older children and adolescents. The work that has been done suggests that genetic influences become more important over the course of development, and that heritability estimates are significantly higher for adolescents than for younger children (Hayiou-Thomas, Dale, & Plomin, 2012; Hoekstra, Bartels, & Boomsma, 2007). In the TEDS sample, we found that individual-differences heritability at age 12 years for a latent factor of four receptive measures, tapping both language structure and higher order language skills, was .59 (Dale, Harlaar, Hayiou-Thomas, & Plomin, 2010; Hayiou-Thomas et al., 2012). At the low extremes, we have found similar levels of heritability (*h*^2^_g_ = 60; Haworth et al., 2009), and we are not aware of other samples that have examined the heritability of LI at similar ages.

## Persistence of Early LI and Diagnostic Criteria: Etiological Evidence

Turning to the question of persistence versus transience of early language delay, previous work from TEDS focused on children with language delay (bottom 10% on expressive vocabulary) at 2 years of age and assessed their outcomes at 4 years (Dale et al., 2003). The early delay group had lower mean scores on expressive language measures at 4 years, and just under half the group (40%) met the criterion for LI status at 4 years. Although language delay at 2 years clearly posed a high risk for language difficulties that persisted to 4 years, the prediction for transient versus persistent difficulties was not strong enough to be of clinical utility. The severity of the language delay did not significantly improve the prediction of outcome, nor did the inclusion of other risk factors such as male gender, low maternal education, and history of ear infections. However, whereas it was difficult to differentiate between the transient and persistent groups at a phenotypic level, there was an interesting distinction between them at an etiological level: Transient language delay was largely environmental in origin, whereas persistent language difficulties were significantly heritable (Bishop, Price, Dale, & Plomin, 2003). A further noteworthy aspect to the finding of different etiology for transient and persistent early language difficulties was the role of parental concern and professional involvement. In the Bishop et al. (2003) study, outcome at 4 years was defined either on the basis of the verbal score on the MacArthur-Bates Communicative Development Inventories (MCDI; Fenson et al., 1994) or on whether the parents were concerned about their child’s language development and had sought professional help. It was only for this latter group that early language delay was substantially heritable (*h*^2^_g_ = .41), whereas for 2-year olds whose parents had not gone on to seek professional help, the heritability of early language delay was close to 0 (Bishop et al., 2003).

The importance of the criteria used to ascertain cases—specifically, whether psychometric measures of verbal ability are used as compared to parental concern and clinical involvement—was further supported by a subsequent study that was carried out with a subsample of 1,600 children from TEDS, who received an in-depth assessment of speech, language, and nonverbal skills at 4½ years of age. A “psychometric” definition of specific language impairment (SLI) was based on these assessments, and heritability for this group was estimated to be surprisingly low: .18 as compared to previous reports in the literature as high as approximately .90 (Bishop, North, & Donlan, 1995; DeThorne et al., 2006; Lewis & Thompson, 1992; Tomblin & Buckwalter, 1998). However, when the TEDS data were reanalyzed such that language status was based on referral to speech and language services, the heritability of SLI was extremely high (.90), which was in line with previous findings. Moreover, the phenotypic characteristics of the referred subsample differed from the partially overlapping psychometric SLI group. That is, the children who were referred for speech and language services had significantly poorer scores on the speech measures than the children who were not referred for services. Thus, it appears that parental and professional concern is more likely to be aroused by speech difficulties than by isolated difficulties in vocabulary and/or grammatical skills—a conclusion that is consistent with previous literature on factors leading to referral (Zhang & Tomblin, 2000). Critically, it is these speech deficits that appear to be under particularly strong genetic influence, and not “pure” language problems, which appear to be influenced to a greater degree by environmental factors (Bishop & Hayiou-Thomas, 2008).

## Etiology of Continuity and Change Between Early Childhood and Adolescent Language

Age 12 years provides an appropriate developmental milestone for characterizing persistent versus transient LI. By this age, there is substantial variation not only in the basic repertoire of language structures—phonology, lexicon, and syntax—but also in most of the more advanced aspects of language that characterize adult language competence, such as use of figurative language, inferential cohesion, decontextualized language use, ambiguity and humor, and metalinguistic awareness (Nippold, 2007). These new skills are essential for successful academic learning, which in turn is a strong predictor of vocational attainment. Thus, LI at 12 years of age is highly likely to have continuing impact on children’s outcomes.

In order to construct a full understanding of the development of the language system, it is important to take long-term developmental changes into account. From an etiological perspective, examining the level of genetic and environmental contributions at different ages is a useful starting point but should ideally be supplemented by an examination of the extent to which the *same* genetic and environmental factors play a role at different points in development. At the level of individual differences across the full distribution, genetic effects seem to play an important role in explaining phenotypic stability in language skills, at least over a 1-year time window, both in the preschool years (Dionne, Dale, Boivin, & Plomin, 2003) and even more so in middle childhood (DeThorne, Harlaar, Petrill, & Deater-Deckard, 2012).

In a long-range analysis of the full range of individual differences in language seen in the TEDS sample, we showed that although there was significant genetic continuity between early (2, 3, and 4 year) and adolescent (12 year) language, there also seemed to be evidence for new genetic factors coming into play (Hayiou-Thomas, Dale, & Plomin, 2012). This could reflect different sets of genes being turned on and off during the onset of adolescence (Pickles et al., 1998) and would be consistent with the moderate genetic correlation of .38 between early and adolescent language skills (i.e., the extent to which the same genetic factors affect variability in language at both ages) and a bivariate heritability of .32 (i.e., the proportion of the overall association between early and 12-year language that can be attributed to genetic factors operating at both ages). In contrast to the genetic results, although shared environmental factors were largely the same across the two age points, they played a much reduced role in the older children. An intriguing finding was a modest but significant increase in the role of unique (nonshared) environmental effects on adolescent language, which may reflect children’s increasing tendency to seek out, or be drawn into, a “niche” that is unique to them and not shared with their siblings (Hayiou-Thomas et al., 2012). In the current article, we aim to examine similar issues with respect to children who are at the low extremes of language ability.

In the present study, we children’s primarily expressive language skills at age 4 years and receptive language skills at age 12 years. In early childhood, expressive language difficulties are more likely than poor comprehension to trigger parental concern and referral to professional speech-language services—and consequently a diagnosis of LI (Zhang & Tomblin, 2000). The impact of poor comprehension becomes more apparent in older children who are learning to read. According to the well-established simple view of reading (Gough & Tumner, 1986), reading comprehension builds on the twin pillars of decoding and oral language comprehension. Children with poor receptive language skills, many of whom may not have been identified as having LI, are particularly likely to struggle with reading comprehension (Nation, Clarke, Marshall, & Durand, 2004). Thus, our focus on expressive language at 4 years and receptive language at 12 years has good ecological validity with respect to current clinical practice. Furthermore, although systematic correlations of expressive and receptive abilities have not been conducted for many aspects of language, they have been explored for vocabulary. At age 12 years, the receptive vocabulary measure, the Peabody Picture Vocabulary Test—Third Edition (PPVT-III; Dunn & Dunn, 1997), and the expressive vocabulary measure, the Expressive Vocabulary Test (EVT; Williams, 1997), are correlated above .8, suggesting that at least for this aspect of language, receptive and expressive skills are very closely related by early adolescence. Nonetheless, we acknowledge that in an ideal design, both expressive and receptive skills would have been assessed longitudinally.

## Research Questions

We asked the following questions at the phenotypic level:

1. How predictive is LI at age 4 years of LI at age 12 years? We examined this issue in terms of both (a) LI status at 12 years and (b) below-average language scores, but not LI, at 12 years. This was an exploratory analysis, and in the absence of comparable previous research, we did not have specific a priori predictions regarding the magnitude of stability over this long developmental span.
2. Does the prediction of LI from age 4 to 12 years differ for alternative definitions of LI at 4 years? Specifically, is there a difference for LI based on low expressive vocabulary and syntax (psychometric LI) as compared to LI based on consultation with a professional (parent referral) or LI based on both psychometric and referral criteria? As this was also an exploratory analysis of a question that has not been previously addressed, we did not have a priori predictions regarding this comparison.
3. What is the nature of parental concern for the alternative definitions of LI at 4 years, in terms of expressive language, receptive language, or speech difficulties? We hypothesized, based on previous literature, that parents would identify speech difficulties and poor expressive language as areas of concern more frequently than poor receptive language skills.

We asked the following questions at the level of genetic and environmental etiology:

1. What is the etiology of LI at 4 years? We compared the etiology of the three diagnostic groups outlined earlier (i.e., psychometric LI, parent referral, and both psychometric and parent referral). Based on previous findings (Bishop & Hayiou-Thomas, 2008), we predicted that parent referral would be more heritable than psychometric LI.
2. Does the etiology of LI at 4 years differ depending on whether language difficulties are still apparent at 12 years? We predicted that there would be greater heritability for persistent LI than transient LI, based on findings at earlier ages (Bishop et al., 2003).
3. What is the etiology of LI at 12 years? Does this differ depending on whether language difficulties were also present at the age of 4 years (persistent LI) or are late emerging at age 12 years (late-onset LI)? We predicted, as above, that persistent LI would be more heritable than late-onset LI.
4. What is the etiology of the relationship between LI at 4 years and LI at 12 years? Based on previous work on individual differences across the full range of ability (Hayiou-Thomas et al., 2012), we predicted both genetic and environmental contributions to this relationship.

## Method

### Participants

The sampling frame for the present study was TEDS, which is a longitudinal study of twins born in England and Wales in 1994, 1995, and 1996 (Oliver & Plomin, 2007; Trouton, Spinath, & Plomin, 2002). After checking for infant mortality, all families identified by the UK Office for National Statistics as having twins born in these years were invited to participate in TEDS when the twins were approximately 18 months old. The twins had been assessed on measures of language, cognitive, and behavioral development at 2, 3, 4, 7, 9, 10, and 12 years of age, using a variety of methods, including parent questionnaires, telephone testing, and web-based assessment.

Twin pairs were excluded where either member of the pair had any major medical or perinatal problems, documented hearing loss, or organic brain damage. Zygosity was determined in same-sex twin pairs by a well-validated parental questionnaire that was completed at 2, 3, and 4 years (Price et al., 2000), with follow-up testing of polymorphic deoxyribonucleic acid (DNA) markers in uncertain cases. Participants for the 12-year study were selected on the basis of previous contributions to data collection in order to maximize the sample size for longitudinal analysis (*N* = 3,979 twin pairs). The sample for the current study was limited to twin pairs with complete data on language measures at ages 4 and 12 years. In all selected families for the current study, English was the only language spoken at home. The current study was based on the resulting sample of 2,923 twin pairs: 1,075 monozygotic (MZ), 975 dizygotic same sex (DZss), and 873 DZ opposite sex (DZos) pairs. The genetic analyses used data from same-sex twin pairs only.

The TEDS sample has continued to be reasonably representative of the UK population with respect to ethnicity, maternal education and employment, and paternal employment (see Haworth, Davis, & Plomin, 2013, for an overview of sample representativeness), although by adolescence, the sample has somewhat higher maternal education and a higher proportion of White families than at study entry. Specifically, in the present sample, the proportion of mothers with at least A-level (university-entrance) qualifications was 45.5%, and the sample was 95.8% White; these compare with 32% and 93%, respectively, in the UK population (Walker, Maher, Coulthard, Goddard, & Thomas, 2001). In addition, the present sample, with data available at 12 years, was not significantly different in standardized age 4 verbal score, which is the score that was used to identify psychometric LI, to the remainder of the sample, −.003 versus .011; *t*(13932) = −.67, *ns*.

### Measures

#### 4-Year Measures

##### Vocabulary

The children’s expressive vocabulary was assessed at 4 years of age by parent report, using an extension of the MCDI that included 48 new words chosen on the basis of literature review and pilot testing. Parents were asked to complete a checklist, indicating which words their children could say (disregarding pronunciation errors).

##### Syntax

The MCDI also includes a measure of children’s sentence complexity. For the extended version used in this study, the parents were asked to indicate on a scale of 1–6 a global rating of the complexity of their child’s language, from not yet talking to talking in long and complicated sentences (see Dale et al., 2003, for the complete wording of this measure). The vocabulary and syntax measures were combined to form a composite language measure. This measure correlates well (*r* = .50) with a composite measure of seven directly assessed standardized language tests that were administered to a subsample of twins at age 4;5 (years;months).

##### Parental concern and professional involvement

As part of a general questionnaire about their children’s development, the parents were asked to indicate whether they had any concerns regarding their children’s language and communication development, and whether they had sought help from a family doctor, speech-language pathologist, or other professional. The parents were also asked to indicate the nature of the language difficulty: (a) language is developing slowly, (b) hard for other people to understand him/her, (c) does not seem to understand other people, (d) pronounces words poorly, (e) does not hear well, or (f) stutters.

#### 12-Year Language Measures

At 12 years of age, the participants were assessed on a web-based set of four language measures, all of which are subtests of well-established published test batteries whose manuals report details of test validation and reliability.^1^ Testing was self-paced, with twins completing the tests individually under parental supervision, and with telephone support from the TEDS team at the beginning of testing and as needed until completion of the battery. Audio streaming was provided for the spoken language stimuli in all of the tests. Further details regarding the development of the webbased battery and the testing procedures are reported in Haworth et al. (2007).

##### Vocabulary

The Vocabulary Multiple Choice subtest of the Wechsler Intelligence Scale for Children—Third Edition as a Process Instrument (WISC–III–PI; Wechsler, 1992) was used as a measure of vocabulary. This well-established published measure has excellent reliability and stability (test manual split-half *r* = .80–.89 [TEDS a = .88]; test–retest *r* = .82–.88 for ages 7–12 years). It also has good criterion- related validity (correlations with other tests of language and reading skills range from .55 to .87) and discriminates well between groups of children independently classified as having high or low levels of ability.

##### Nonliteral semantics

The Figurative Language subtest of the Test of Language Competence—Expanded Edition, Level 2 (TLC–E; Wiig, Secord, & Sabers, 1989) was used as a measure of semantics. This subtest assesses the interpretation of idioms and metaphors; correct understanding of such nonliteral language requires rich semantic representation as well as an awareness of the ambiguity of many expressions between their literal and figurative meaning. In this subtest, the child is presented with a sentence orally and is required to choose one of four answers, presented in both written and oral forms (test manual a = .67 [TEDS a = .67]; test–retest *r* = .73; criterion-related validity for the overall TLC–E evidenced by correlations of .62–.78 with comparable measures of language ability; 96% sensitivity for identifying individuals with language learning disorders).

##### Syntax

Syntax was assessed using the Listening Grammar subtest of the Test of Adolescent and Adult Language—Third Edition (TOAL-3; Hammill, Brown, Larsen, & Wiederholt, 1994). In this subtest, children are required to select two sentences that have nearly the same meaning from a set of three options. The sentences were presented orally only (test manual α = .94 [TEDS a = .94]; test-retest *r* = .81; criterion-related validity for overall TOAL–3 evidenced by correlations of .59–.83 with comparable measures of language ability; 89% sensitivity for identifying individuals with language learning disorders).

##### Pragmatics

The Making Inferences subtest of the TLC–E, Level 2, was used to test pragmatics. In this subtest, the child is required to make permissible inferences on the basis of existing but incomplete causal relationships in the context of short paragraphs presented orally. The child chooses two of four responses, presented in both written and oral form, that best explain what could have happened (test manual a = .71 [TEDS α = .58]; test–retest *r* = .54; summary of validity information for TLC–E as before).

For the purposes of the current study, we used a composite score averaging *z* scores of the four individual measures. This was based on previous work showing that all four measures had high loadings on a common factor (.61–.71), and that there was substantial etiological as well as phenotypic overlap among these measures (Dale et al., 2010).

#### Additional Family Measures

At entry in the study, the mothers provided information on their educational attainment (“qualifications”). These were scored on an 8-point basis, ranging from 0 = *none through 4* (A-level exams taken at age 18 by students anticipating university education) to 7 = *undergraduate degree* and 8 = *postgraduate degree*.

At twin age 9 years, the parents provided information about family history of early language and/or reading difficulties. The family history variable was coded as one if any first degree relative (i.e., mother, father, older brother, older sister) was reported as having either type of difficulty; otherwise, it was coded as zero.

#### Definitions of LI

We compared two criteria for LI at 4 years of age. Psychometric LI was defined as scoring lower than −1.25 *SD*s below the sample mean (equivalent to the lowest ~10% of the sample), and parent referral was defined on the basis of referral to a professional following parental concern about a child’s language and communication skills. We compared three mutually exclusive categories of LI: psychometric LI without professional involvement (hereafter, psychometric LI), parent referral without qualification as LI on the basis of verbal score (hereafter, parent referral), and both psychometric and parent referral (hereafter, both). Note that the term parent referral does not refer to classification as LI on the basis of a qualified clinician, but only on the parent’s choice to consult with a professional at some point.

At age 12 years, LI was defined on the basis of a score lower than −1.25 *SD*s below the sample mean on the 12-year language composite measure described earlier. Children with LI at 4 years and also at 12 years were classified as persistent LI; those with LI at 4 years but not at 12 years were classified as transient LI. These classifications were made separately for initial psychometric LI and parent referral.

At both 4 and 12 years of age, proband selection was based on language scores that were corrected for age but not sex in order to include a representative distribution of boys and girls.

### Genetic Analyses

The genetic analyses were based on the twin design, which capitalizes on the fact that identical (MZ) twins share 100% of their varying DNA whereas fraternal twins (DZ) share on average 50%, just like any other sibling pair (Plomin, DeFries, McClearn, & McGuffin, 2008). Greater similarity on a trait or disorder between MZ as compared to DZ twins is indicative of a genetic contribution to that trait. “Shared environment” refers to environmental factors that contribute to within-pair similarity for both MZ and DZ pairs, whereas “nonshared” or “unique” environment refers to factors that are unique to one member of a twin pair and thus reduce within-pair similarity. Measurement error, because it is assumed to be uncorrelated between members of a twin pair, is included in the nonshared environment parameter.

We used two different analytic approaches to address our research questions because our data included both continuous and categorical variables. Proband-wise concordance and liability threshold models were applied to the categorical data (parental concern and professional involvement at 4 years), whereas DeFries-Fulker extremes analysis (DF; DeFries & Fulker, 1985, 1988) was used for the continuous data.

#### Proband-Wise Concordance

Proband-wise concordance rates were estimated for each definition of LI at 4 and 12 years to indicate the probability that the co-twin of an affected twin would also be affected. It is calculated as 2C/(2C + D), where C is the number of concordant pairs (each of which has two probands) and D is the number of discordant pairs (which have only one proband). If the concordances are generally high, this indicates familiality; further, if MZ twins have higher concordance rates than DZ twins, genetic influence is suggested.

#### Liability Threshold Modeling

The liability threshold model, which is a natural extension of biometric models for quantitative traits, is widely used in the field of genetics to analyze concordance data (Sham, 1998). The model assumes an underlying continuous liability that has a normal distribution, with a mean of 0 and a variance of 1 in the general population. If the liability to a disorder is quantitative rather than categorical, the disorder is assumed to be present in all individuals whose liability is above a certain threshold value and to be absent in all other individuals. The value of the threshold can be estimated from the population frequency of the disorder. The liability is not measured directly but is estimated from the observed categorical data. For the purposes of this study, the data from the entire twin sample were organized into 2 × 2 contingency tables, where some cells represent pairs in which both twins are unaffected and some represent pairs in which both twins are probands, as well as two discordant cells where twin one or twin two is a proband. These data can be used to quantify genetic and environmental sources of variation in liability in the population. In this study, a structural equation model was fit to the contingency tables by maximum likelihood, using the Mx software program (Neale, Boker, Xie, & Maes, 2002) to estimate additive genetic, shared environmental, and nonshared environmental parameters (Neale, 1997).

#### DF Extremes Analysis

DF extremes analysis takes advantage of continuous measurement of individuals’ ability rather than a purely categorical approach (DeFries & Fulker, 1985, 1988). Probands are diagnosed categorically as in concordance estimates (although the categorical boundary can simply be a low score on the trait of interest), but instead of assigning affected or not-affected status to the co-twins, their quantitative scores on the trait of interest are calculated. Prior to analysis, individuals’ scores are transformed to account for mean differences between the MZ and DZ probands. This is done by dividing both proband and co-twin mean scores by the proband mean score separately for each zygosity. The result is a proband mean of 1.0; by definition, the standardized scores for the population have a mean of 0.

The basic univariate DF extremes model (DeFries & Fulker, 1985, 1988) uses multiple regression to estimate the differential regression to the population mean of the MZ and DZ co-twins of affected probands. The mean MZ and DZ co-twin scores, specifically, the extent to which they are below the population mean, index the similarity of the co-twins to the probands (group-differences familiality). The difference between the MZ and DZ co-twin means is an index of group heritability and indicates the extent to which genetic factors contribute to the difference between the probands as a group and the normal population. Twin resemblance that is not explained by genetic factors, referred to as group shared environment (*c*^2^_g_), can be estimated by subtracting group-differences heritability from group-differences familiality (the transformed MZ co-twin mean). Residual influences are attributed to the nonshared environment. The basic multiple regression model is as follows: C = b_1_P + b _2_R + A, where C is the co-twin’s predicted score, P is the proband’s score, R is the coefficient of the relationship, A is the regression constant. b_1_ is the partial regression of the co-twin’s score on the proband’s score and is a measure of twin resemblance independent of zygosity, and b _2_ is the partial regression of the co-twin’s score on the coefficient of relationship. As an approximate rule of thumb, group heritability estimates smaller than .25 can be considered “small,” those between .25 and .50 “moderate,” and those greater than .50 “large.”

The DF extremes model can be extended to test the significance of a difference in heritability between two groups (Castles, Datta, Gayan, & Olson, 1999). This is done by including a term (S) for the variable differentiating the two groups (e.g., outcome status at age 12, when comparing transient and persistent LI). The resulting equation is C = b_1_.P + b_2_.R + b_3_.S + b_4_.P.S + b_5_.R.S. If the regression coefficient for the R.S term, b5, is significant, this indicates that there is a reliable interaction, such that the group heritability depends on the level of S. The DF extremes model can also be extended to the bivariate case (more fully explained in Purcell et al., 2001), in which proband selection based on X is related to co-twin performance on Y. The regression coefficient here indicates the degree to which genetic factors are responsible for the lowered Y scores of low-X probands. In the current study, this is the degree to which the factors contributing to LI status at 4 years are responsible for lower language scores at 12 years. The ratio of the bivariate regression coefficient to the phenotypic association is also informative, as this indicates the extent to which the overall association between X and Y at the extremes can be explained by shared genetic factors. The phenotypic association is expressed in terms of the phenotypic group correlation, which is the ratio of the proband mean on the standardized unselected variable (e.g., 12-year language score) to the proband mean on the standardized selected variable (e.g., 4-year language score; Oliver, Dale, & Plomin, 2004).

## Results

### Phenotypic Analyses

In order to preserve the independence of data, the phenotypic analyses were based on a random selection of one twin from each pair. In addition, the analyses included DZos twin pairs, who were not included in the genetic analyses. Descriptive information about the full sample and the subgroups identified as LI by the various, mutually exclusive definitions at age 4 years are summarized in Table 1. As expected from the definition, children in the psychometric LI group had lower language scores than those in the parent- referral group, but children in the both group scored the lowest. Males were predominant when the parent-referral definition was used; their proportion rose to 70% in the group who met both criteria.

The modest overlap between the two definitions of LI is apparent from the numbers in Table 1. Of the 518 children who met at least one of the criteria, only 110 (21.2%) met both. This substantial difference is an important motivation for separate analyses of the outcome of early LI by the two definitions. The groups were also compared with respect to maternal education and to family history of language and/or reading difficulties. The most notable aspect of that comparison concerns the parent-referral category, which was characterized by above average maternal education but the highest rate of family history. In contrast, the lowest mean maternal education was found in the psychometric LI group. A one-way analysis of variance (ANOVA) comparing the four groups confirmed an overall difference in maternal education, *F*(3, 2854) = 7.17, *p* < .001. Follow-up Bonferroni multiple comparisons confirmed that maternal education was significantly lower for the psychometric LI group than for the neither group, as well as significantly lower for the psychometric LI group than for the parent-referral group. A parallel omnibus chi-square comparison of the four groups with respect to family history confirmed overall differences, c^2^(3) = 7.89, *p* < .05. However, none of the follow-up comparisons of the individual groups reached significance.

#### How predictive is LI from age 4 to age 12? Does this differ for alternative definitions of LI at age 4?

Table 2 compares the groups with respect to outcome at age 12 years. A one-way ANOVA comparing the four groups with respect to 12-year language confirmed an overall difference, *F*(3, 2918) = 37.8, *p* < .001. Follow-up Bonferroni comparisons confirmed that (a) twins in the psychometric LI group and the both group at 4 years scored lower than twins in the neither group at 4 years, (b) twins in the psychometric LI group scored lower than twins in the parent-referral group, and (c) twins in the both group scored lower than twins in the parent-referral group.

At the level of categorical classification, the stability, that is, the persistence of early LI, was only modest, with 11%–29% of each group meeting the criterion for LI at age 12 years. An omnibus chi-square comparison of the four groups confirmed an overall difference, c^2^(6) = 68.9, *p* < .001. Follow-up chi-square comparisons confirmed that twins in the psychometric LI group and in the both group were more likely to be LI at 12 years than twins in the parent-referral group. In addition, there was also an elevated proportion of children who scored below the mean at age 12 years but did not qualify as having LI. Overall, stability was higher for children in the psychometric LI group than for those in the parent-referral group, and stability was highest for the children in the both group. There was little evidence for a difference in stability for males and females.

The likelihood ratios (LRs) reported in the final column in rows 2 and 4, although comparable to those found in many epidemiological settings, are far from being useful for individual prediction. For example, Dollaghan (2007) suggested that a positive LR of 3 is only moderately positive (“suggestive but insufficient to diagnose disorder”) and that the ratio should be at least 10 to be viewed as very positive (“very likely to have come from a person with the disorder”). Note that the ratio reported for the parent-referral group is less than 1, reflecting the fact that LI at 12 years is less likely for this group than for the sample as a whole.

#### What is the nature of parental concern for the psychometric LI group versus the parent-referral group?

We examined the nature of parental concerns for children meeting the criteria for psychometric LI, the parent-referral group, or both. Table 3 characterizes these three groups, comparing the proportion of children whose parents indicated concern about their child’s expressive language skills (“developing language slowly”), receptive language skills (“doesn’t understand”), or speech (“pronounces poorly”).

The rate of concerns for the psychometric LI group was very low,^2^ similar to the overall sample average. The much higher rates for the parent-referral group indicate clearly that speech difficulties were the most frequent trigger for parental concern, followed by expressive language; only a very small fraction of responses indicated difficulties in the children’s receptive language skills. The group of children meeting the criteria for both psychometric LI and parent referral had the highest overall rate of reported concerns, as well as the broadest profile: As in the other groups, the most frequent concern, for nearly 2/3 of this group, related to speech difficulties. A strikingly large proportion of this group—more than half—had parent-reported difficulties in expressive language. Finally, although a relatively small proportion (10%) of the children’s parents were concerned about their receptive language difficulties, this was a notably greater proportion than in the other groups.

In summary, the most frequent trigger for parental concern, consistently across all groups, was speech difficulties, followed by slow development of expressive language skills; only a very small number of parents indicated any concern about their child’s receptive language ability.

### Genetic Analyses

#### What is the etiology of LI at 4 years? Comparing psychometric and parent-referral definitions

We compared the univariate heritability and environmentality estimates for LI at age 4 years for the diagnostic groups outlined earlier. Table 4 presents the results obtained by the DF extremes analysis for the diagnostic categories based on parent-reported verbal ability, psychometric LI, and both psychometric LI and parent referral.^3^ When verbal ability was the only criterion used in the definition of LI (row 1 in Table 4), the transformed co-twin mean for the MZ pairs was very high (.88), indicating a high level of familiality. It was somewhat lower for the DZ pairs (.69). This corresponds to moderate heritability for both of these definitions of LI (*h*^2^_g_ = .37), and a similar level of shared environmental influence (*c*^2^_g_ = .51). The nonshared environment made a modest contribution (*e*^2^_g_ = .12), which also includes measurement error. A rather different result emerged when the definition of LI was based on both psychometric ability and clinical involvement: The MZ transformed co-twin mean (.90) was more than twice as large as the transformed DZ mean (.33), suggesting that for this subgroup, LI status was entirely dependent on genetic factors (*h*^2^_g_ = 1.00, *c*^2^_g_ = .00). The size of the difference between the MZ and DZ co-twin means was also consistent with the presence of nonadditive genetic effects.

This pattern of results suggests a different etiology for LI that is diagnosed on the basis of psychometrically evaluated verbal ability as opposed to parental concern and clinical involvement. In order to test this possibility more directly, we carried out a series of liability threshold analyses, comparing our three diagnostic categories (Table 5).^4^ The large difference between the MZ and DZ twins in terms of both proband-wise concordances and the tetrachoric correlations suggests high heritability for the two groups incorporating parent referral in the definition (rows 2 and 3). This is in contrast to the relative similarity of the MZ and DZ concordances and correlations for the psychometric LI group (row 1), which points to low heritability. This conclusion is confirmed by the results of the liability threshold models, which show substantially higher heritability estimates for the parent-referral and both groups (*a*^2^ = .64–.73) as compared to the psychometric LI group (*a*^2^ = .18). Note that the confidence intervals overlap slightly, making this difference marginally significant with this sample size.

The pattern of results for the environmentality estimates reveals even more dramatic differences in the etiology of the psychometric LI group versus the parent-referral group. Shared environmental factors appeared to be the dominant influence on psychometric LI (*c*^2^ = .77), whereas they were not significantly different from 0 for the parent- referral group (*c*^2^ = .04), with the confidence interval crossing 0. In addition, there was no overlap in the confidence intervals for these two groups. By contrast, nonshared environmental influences, though modest, exerted a significantly greater influence in the parent-referral group than in the psychometric LI group (*e*^2^_g_ = .23 compared to .04).

#### Does the etiology of LI at 4 years differ for persistent versus transient LI?

We examined the etiology of LI at 4 years as a function of whether or not it persisted to age 12 years. Because the phenotypic analyses suggested that LI at 12 years was predicted better by psychometric LI than parent referral at 4 years, we focused these analyses on the former category. Persistent LI was defined on the basis of meeting the criteria for psychometric LI at 4 years and scoring lower than −1.25 *SD*s below the population mean on the language composite at 12 years. Transient LI was defined as meeting the criteria for psychometric LI at 4 years and scoring greater than −1.25 *SD*s below the population mean at 12 years.

The results of the DF extremes analysis comparing these two groups are presented in Table 6. The transformed co-twin means for the MZ and DZ pairs were similar for both transient and persistent LI, reflecting similar heritability (*h*^2^_g_ = .38 and .40, respectively) and environmentality estimates (*c*^2^_g_ = .47 and .55, respectively). The overlapping confidence intervals indicate that the differences in etiology between these two groups were not significant.

#### Comparing the etiology of transient/persistent LI and psychometric LI/parent referral

**Figure 1:**
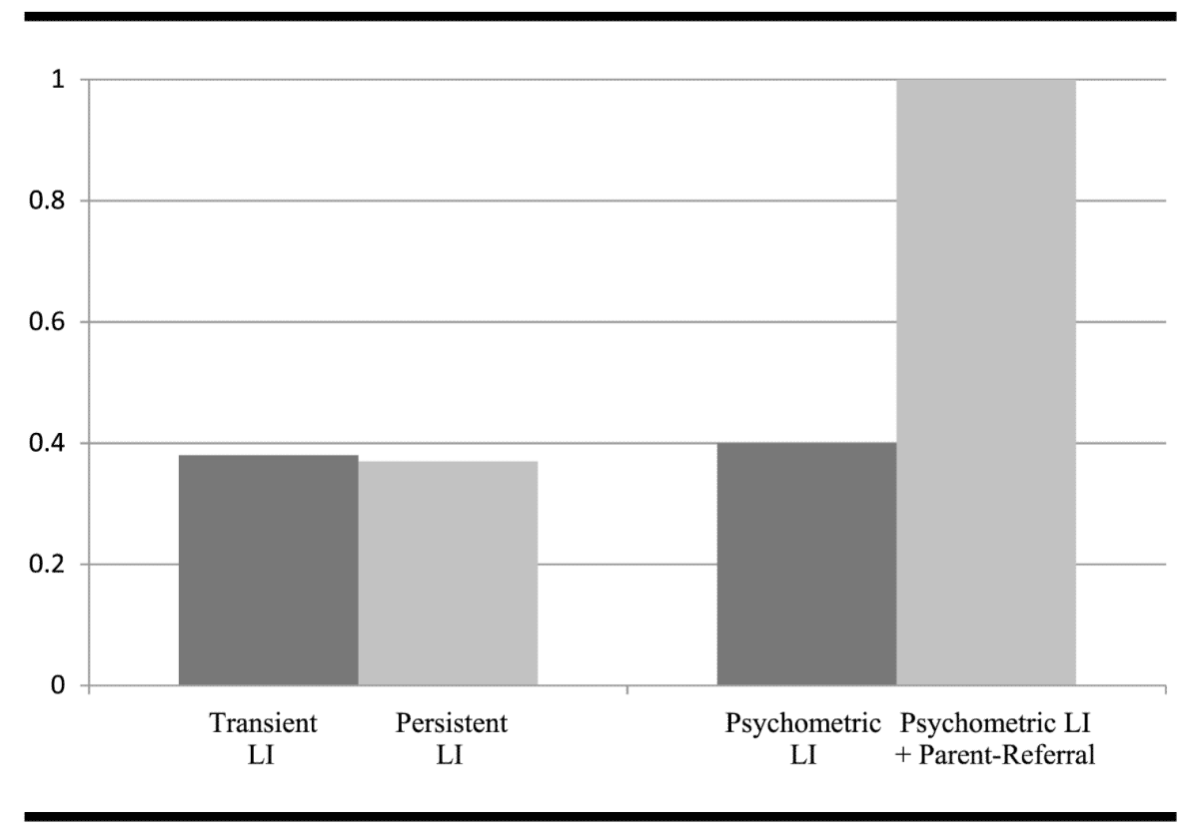
Heritability of language impairment (LI) at 4 years: Comparing transient LI versus persistent LI, and psychometric LI versus combined psychometric LI and parent referral.

The analyses presented earlier strongly suggest that substantial heritability for early LI is related not to whether the LI turns out to be persistent or transient, but by whether the diagnosis is driven by clinical involvement rather than a psychometric measure of verbal ability. We carried out two augmented DF extremes analyses, as described earlier, to test the statistical significance of this pattern of heritability. These analyses confirmed that there was no significant interaction with persistence (b = .08, SE = .18, *p* = .64): that is, the heritability of verbal scores at 4 years was not significantly different for the group of children who go on to have language difficulties at 12 years and those who do not. By contrast, there was a significant interaction with clinical involvement (b = .77, SE = .16, *p* < .00), such that the heritability of verbal scores at 4 years for the group who has seen a clinician is significantly higher than for the group who has not seen a clinician. This interaction is illustrated in Figure 1.

#### What is the etiology of LI at 12 years? Does it differ for persistent versus late-onset LI?

LI at 12 years was defined as scoring lower than −1.25 *SD*s below the population mean on the 12-year language factor. We examined the heritability of LI at this age and compared subgroups meeting criteria for persistent LI (psychometric LI at 4 years and LI at 12 years of age) and late-onset LI (LI at 12 years, but no psychometric LI at 4 years of age), as well as the combined group meeting either of these criteria.^5^ The results of these analyses, reported in Table 7, indicated similar etiology for all three definitions of LI at 12 years. The transformed MZ co-twin means were in a similar range (.65–.75), as were the DZ co-twin means (.48–.62). The heritability estimates were moderate and did not differ significantly for the three groups (*h*^2^_g_ = .25–.35). The shared environmental estimates were similar to the heritability estimates and were also similar for the three groups (*c*^2^_g_ = .31–.50).

These estimates are quite close to the heritability and shared environmentality estimates for psychometric LI at 4 years. There is an interesting divergence from the 4-year results in the nonshared parameter estimate, which appears to be as important as shared environment for LI at 12 years (*e*^2^_g_ = .25–.35). However, the nonshared environment parameter includes measurement error, and so it must be inter- preted cautiously.

#### What is the etiology of the relationship between LI at 4 years and LI at 12 years?

A comparison of the heritability and environmentality estimates of the univariate analyses of LI at 4 and 12 years suggests a broadly similar pattern of etiology, with the possibility that nonshared environmental influences become more important at later ages. However, the univariate analyses do not allow a direct examination of the etiology of the *relationship* between LI at 4 and 12 years. A bivariate DF extremes analysis was carried out to look into this issue, examining the influence of LI status at 4 years on language skills at 12 years. The low MZ (227 pairs) transformed co-twin mean of .28 is indicative of a relatively modest overall relationship between LI at 4 years and language at 12 years and is consistent with the phenotypic results reported earlier. The similarly low DZ (116 pairs) transformed co-twin mean of .22 reflects the small, and statistically nonsignificant, bivariate heritability estimate of *h*^2^_g_ = .12, 95% CI [−.13, .37], which is similar to the small, nonsignificant, shared environmentality estimate of *c*^2^_g_ = .16, 95% CI [−.06−.37]. That is, to the extent that LI at 4 and 12 years are related at all, the point estimates (bearing in mind the wide confidence intervals) suggest that this is due to both the genetic and shared environmental factors that they have in common.

## Discussion

Our results, which are consistent with previous findings, showed that having an expressive LI at 4 years of age poses an increased risk for having LI—or at least below- average language skills—at 12 years, but that there is also a large amount of variability in outcome. Approximately one third of the children with LI at 4 years went on to have average or above-average language skills at 12 years, whereas one third of the children meeting the LI criteria at 12 years had no apparent difficulties at 4 years. Why is the overall level of stability only moderate? It may be that there is a relatively high degree of spontaneous resolution of language difficulties between the ages of 4 and 12 years, as has often been reported for the early years of language acquisition (e.g., Rescorla, 2002). An alternative possibility is that some children with early LI received effective treatment (or other educational intervention) that ameliorated their early difficulties. It is also possible that early language difficulties manifest later on in the form of literacy problems rather than obvious deficits in oral language (e.g., Stothard et al., 1998). This is an issue we plan to investigate in future work using the TEDS data set. In addition to these substantive possible explanations for our finding of moderate stability of LI between 4 and 12 years of age are methodological issues that need to be considered and that we expand on in the Limitations section.

One of the issues we were particularly interested in examining was the diagnostic criterion that was used to identify LI at 4 years; specifically, whether a psychometric as opposed to parent-referral definition would be a better predictor of long-term difficulties. Our data suggested that children in the psychometric LI group are considerably more likely to experience long-term language difficulties, whereas children in the parent-referral group are relatively unlikely to experience such difficulties.

Two methodological provisos should be added to this conclusion. First, LI at 12 years was defined on the basis of a child’s performance on directly assessed language measures, and so it may be unsurprising that it is the directly assessed early language measure that relates relatively better to a later language measure. We do not have professional involvement information at age 12 years, so were not able to examine the issue of whether parent referral at 4 years would be more predictive than psychometric LI of a parent-referral classification at 12 years. Second, the severity of the LI at 4 years was different for the psychometric LI and parent-referral groups. In fact, the ordering of the groups with respect to the likelihood of LI at 12 years exactly matched the ordering by severity of impairment on the verbal score at 4 years; in particular, children who were in the both group had both the lowest scores at 4 years and the greatest likelihood of being LI at 12 years.

An alternative, somewhat speculative explanation for the better predictive power of psychometric LI as compared to parent referral lies in the nature of the parental concern noted for these groups. More than half of children in the parent- referral group were described by their parents as having speech difficulties, whereas only 8% of the children in the psychometric LI group were so described. This suggests that the presence of speech difficulties is particularly likely to arouse parental concern and to lead to referral to professional services. This is consistent with previous work in the field about the factors that lead to referral in young children (Bishop & Hayiou-Thomas, 2008; Zhang & Tomblin, 2000). An added insight from the current study comes from the longitudinal design, which revealed that early language difficulties, even if unnoticed or unremarked, are more likely to be predictive of language difficulties at later ages; in contrast, the difficulties that concern the parents of 4-year-olds—with speech prominent among these—seem to pose relatively little risk for long-term LI, at least as defined by our (receptive) measures.

Thus, at the phenotypic level, the psychometric LI versus parent-referral distinction yielded interesting results with respect to the prediction of long-term language outcomes. It also yielded an interesting distinction in terms of etiology, in that parent referral, based on parental concern and professional involvement, captured a significantly more heritable phenotype than psychometric LI, based on expressive vocabulary and syntactic ability. This replicates and extends our previous work and appears to be a robust finding in that it holds at different ages (2 and 4 years—compare the current study with Bishop et al., 2003), using different measures of verbal ability (MCDI vs. a directly administered battery of language measures—compare the current study with Bishop & Hayiou-Thomas, 2008), different measures of clinical concern (parental concern and professional involvement by age 4 years in the current study and Bishop et al., 2003; and speech-language treatment by age 7 years in Bishop & Hayiou-Thomas, 2008), and different analysis methods (compare DF extremes analysis and liability threshold results in the current study).

In addition to the psychometric LI/parent-referral distinction, we were also interested to see whether transient, persistent, or late-onset LI might have different etiological bases. We found no evidence for this: Although there was a slight trend for greater heritability for persistent difficulties, there were no significant differences in the heritability and environmentality estimates for each of these categories. This finding is counterintuitive, but in fact is consistent with the Bishop et al. (2003) analysis of transient versus persistent delays at 2 years of age: Higher heritability was only reported for persistent delays *that aroused parental concern*. Thus it appears that parental concern—often about speech rather than language—is the marker of a heritable impairment, but not persistence of oral language difficulties, whether the persistence is in the early years (2–4 years) or over a longer time frame (4–12 years), as in the current study.

To summarize, parents and professionals are sensitive to the heritable impairment that is often related to speech difficulties, but this is not predictive of long-term LI. The less heritable, more environmentally driven language difficulties are the ones that are likely to predict long-term language outcomes.

### Etiology of Psychometric LI at 4 and 12 Years: The Role of Nonshared Environment

In line with previous work on the TEDS sample and in the wider literature, we found moderate levels of heritability on psychometric LI at both 4 and 12 years. Environmental factors played a substantial role in LI at both ages, but with a striking difference: At age 4 years, the environmental influence was almost all due to the shared environment, with minimal nonshared environmental effects. At age 12 years, however, the effects of the nonshared environment were as important as those of the shared environment. This seemed to be the case regardless of whether the LI at 12 years was preceded by earlier difficulties (persistent) or not (late-onset LI). Although the nonshared environment parameter includes measurement error, and it is therefore necessary to interpret this estimate with caution, our results are suggestive of an increase in the importance of nonshared environment as a causal factor in LI in early adolescence. Such an increase has also been observed in other domains, such as general intelligence (Plomin et al., 2008), and is consistent with our previous work focusing on individual differences across the whole range of language ability, in which we also observed a rise—though a more modest one—in the role of the non- shared environment at age 12 years.

This pattern suggests that LI in early adolescence is partly influenced by family-level variables such as socioeconomic status (which might include financial resources to obtain treatment, etc.), but also important are individual aspects of experience such as family response to an individual child with LI (specifically, that part of the family response that is itself not genetically driven), peer interactions, quality of treatment, and health issues. A crucial task for future research is to identify those individual child-level variables that make a difference.

### Limitations

There are some general limitations with respect to our measures that should be borne in mind when interpreting our results. These arise partly from the fact that adequate statistical power in twin studies requires very large samples, but this places constraints on the depth of measurement possible. Given those constraints, parent report for young children and web-based testing for adolescents represent the best available methodologies at present. First, our language measure at 4 years was a composite of a single measure of expressive vocabulary and a single measure of syntactic ability, based on parent report. Ideally, we would have had multiple measures assessing different aspects of receptive and expressive language ability. However, our MCDI measure at 4 years correlated well (~.5) with a composite of seven diverse measures of receptive and expressive language administered by an independent tester, for a subset of the children included in this study. At an etiological level, too, the MCDI measure at 4 years showed similar levels of heritability and environmentality to the composite of direct measures (Hayiou-Thomas et al., 2006; Spinath et al., 2004).

In a similar vein, although our predictor measure at 4 years assessed expressive language, our outcome measure at 12 years assessed receptive language. It is possible that a stronger prediction would emerge if the predictor and outcome measures were in the same modality. In this sense, crossing modalities made our current analyses rather conservative, and they may underestimate the true level of prediction from 4 to 12 years. On the other hand, in previous work focusing on individual differences across the whole range of ability, we found very similar results in terms of the relationships between early and later (12 year) language, for the directly assessed receptive measures and a global teacher rating of language ability, which presumably includes a substantial expressive component (Hayiou-Thomas et al., 2012). It seems reasonably likely, therefore, that we would have obtained similar results had we used more extensive measures at 4 years and matched them in terms of modality to the language measures at 12 years. Nonetheless, it should be borne in mind that when we refer to persistence, we mean that some form of LI was present at both time points in the study, but that does not necessarily imply stability in the type of language difficulty experienced: that is an important issue, but is beyond the scope of this study.

Finally, with respect to measures and definitions, we reiterate that when we referred to the parent-referral group, we were referring to parental concern about the child’s language development and whether a professional was consulted. This does not constitute a clinical diagnosis as made by a professional clinician. However, it is worth noting that our findings with respect to the speech and language profile of this group, as well as with respect to the high heritability, do fit with the general picture in the field (Zhang & Tomblin, 2000), again suggesting that it is likely we would have obtained similar results had we had access to a full clinical diagnosis.

A further issue that should be borne in mind when interpreting our results is the use of two different analysis methods: DF extremes analysis, which is suitable for use with continuous data, and liability threshold modeling, which is suitable for use with dichotomous data (or continuous data that have been dichotomized). In the case of psychometric LI at age 4 years, where both approaches were used for the same analysis, DF extremes yielded slightly higher estimates of heritability and lower estimates of shared environment than liability threshold modeling; however, the overlapping confidence intervals indicated that these differences were not significant. More importantly, the pattern of results was highly consistent across analysis methods, with respect to the greater heritability (and negligible shared environmentality) for the parent-referral group as compared to the psychometric LI group at 4 years. This underscores a more general point about the interpretation of heritability and environmentality estimates; namely, that the general pattern of estimated parameters is more interpretable than the exact numerical estimates.

A general point regarding the scope of the current article is that we chose to focus on the language domain for the sake of clarity. However, there is increasing evidence that the etiology of LI overlaps substantially with other domains and disorders, such as dyslexia (e.g., Hayiou-Thomas, Harlaar, Dale, & Plomin, 2010; Pennington & Bishop, 2009) and autism (Dworzynski et al., 2007; Vernes et al., 2008). These cross-domain relationships will be crucial in creating a comprehensive picture of atypical language development.

## Conclusion

Consistent with the previous literature, we found that LI at 4 years was a substantial risk factor for LI at 12 years, although the prediction was not strong enough to be of clinical utility at the individual level. Our most novel findings, however, centered around the contrast between psychometric LI, based on expressive language abilities at 4 years, with parent referral, based on parental concern about a child’s language development at the same age. These two diagnostic criteria for LI differ in some important ways: (a) Psychometric LI is more predictive of poor language performance at age 12 years than parent referral; (b) parent referral is significantly more heritable than psychometric LI; and (c) although psychometric LI (unsurprisingly) reflects poor oral language abilities, the parent-referral group seemed to be particularly likely to have speech difficulties. Thus, it seems that parental concern is more likely to be aroused by speech than by language problems, and this in turn seems to be the marker of a more heritable disorder. However, it is the less heritable disorder—of psychometrically defined early expressive language difficulties—that is the better indicator of long-term language outcome.

## Figures and Tables

**Table 1:** Comparison of classifications of language impairment (LI) at 4 years of age.

Classification	Twins at 4 years		% males in LI group	Verbal score at 4 years		Maternal education		% with family history (1st degree) of language or reading difficulties
	*N*	%		*M*	*SD*	*M*	*SD*	
Full sample	2,923	100.0	44.2	0.05	0.95	4.17	2.02	9.3
Psychometric LI	237	8.1	46.4	−1.79	0.41	3.57	1.92	11.0
Parent referral	171	5.9	54.4	0.13	0.71	4.27	2.12	14.0
Both psychometric LI and parent referral	110	3.8	70.0	−2.08	0.50	3.87	1.93	12.7
Neither	2,405	82.3	42.0	0.33	0.65	4.17	2.02	8.7

*Note*. Verbal score at 4 = age-regressed parent-reported expressive vocabulary *z* score; maternal education = level of maternal qualification, where 0 = none, 1 = General Certificate of Secondary Education (D, E, F, G grade) or equivalent school-leaving qualification, 2 = General Certificate of Secondary Education (A, B, C grade) or equivalent school-leaving qualification, 3 = A-level or equivalent university-entrance qualification, 4 = Higher National Certificate (tertiary vocational qualification), 5 = Higher National Diploma (tertiary vocational qualification), 6 = undergraduate degree, and 7 = postgraduate degree.

**Table 2:** Outcome at 12 years of classification of LI at 4 years.

Classification	Twins at 4 years		Language score at 12 years		% of LI twins at 4 who are LI at 12 (F; M)	% of twins LI at 4 years below mean at 12, but not LI	Likelihood ratio (LR+) for delay at 12 years
	*N*	%	*M*	*SD*			
Full sample	2,922	100.0	0.01	0.99	12.4 (12.4; 12.3)	32.5	1.0
Psychometric LI	237	8.1	−0.44	1.08	24.5 (25.2; 23.6)	37.1	2.3
Parent referral	171	5.9	−0.06	0.97	11.1 (11.5; 10.8)	38.0	0.9
Both psychometric LI and parent referral	110	3.8	−0.65	1.06	29.1 (27.3; 29.9)	40.0	3.0
Neither	2,404	82.3	0.09	0.96	10.5 (10.9; 9.9)	31.3	0.8

*Note*. Language score at 12 = age-regressed composite score from the four receptive language measures.

**Table 3:** Analysis of parental concern by category.

Question	All		Psychometric LI		Parent referral		Both	
	Frequency	%	Frequency	%	Frequency	%	Frequency	%
Expressive language (“Developing language slowly”)	125	4.3	10	4.2	37	21.6	63	57.3
Receptive language (“Doesn’t understand”)	15	0.5	1	0.4	2	1.2	12	10.9
Speech (“Pronounces poorly”)	233	8.0	19	8.0	98	57.3	72	65.5

**Table 4:** Univariate DeFries-Fulker extremes analysis: Etiology of LI at 4 years.

Classification	*N*		Transformed co-twin mean		Group heritability (95% CI)	Group shared environmentality (95% CI)	Group nonshared environmentality
	MZ	DZ	MZ	DZ			
Psychometric LI	226	115	.88	.69	.37 [0.15, 0.59]	.51 [.0.27, 0.74]	.12
Both psychometric LI and parent referral	71	51	.90	.33	>1.00 [0.74, 1.56]	.00	.00

*Note*. MZ = monozygotic; DZ = dizygotic; CI = confidence interval.

**Table 5:** Proband-wise concordance, tetrachoric correlations, and liability threshold model results for LI at 4 years: Direct comparison for the psychometric LI and parent-referral groups.

Classification	*N*		Proband-wise concordance		Tetrachoric correlations		Liability threshold parameter estimates 95% CI		
	MZ	DZ	MZ	DZ	MZ	DZ	*a* ^2^	*c* ^2^	*e* ^2^
Psychometric LI	226	115	.90	.73	.96	.84	.18 [.06-.36]	.77 [.60-.89]	.04 [.02-.07]
Parent referral	112	104	.65	.35	.77	.40	.73 [.30-.85]	.04 [.00-.41]	.23 [.14-.35]
Both psychometric LI and parent referral	71	51	.95	.48	.99	.67	.64 [.34-1.00]	.35 [.00-.66]	.01 [.00-.03]

*Note*. CI = confidence interval; *a*^2^ = heritability; *c*^2^ = shared environmentality; *e*^2^ = nonshared environmentality.

**Table 6:** Univariate DeFries-Fulker extremes analysis for LI at 4 years: Transient versus persistent LI.

Classification	*N*		Transformed co-twin mean		*h*^2^_g_ (95% CI)	*c*^2^_g_ (95% CI)	*e* ^2^_g_
	MZ	DZ	MZ	DZ			
Transient LI	167	88	.85	.66	.38 [0.12, 0.65]	.47 [0.20, 0.78]	.15
Persistent LI	60	28	.95	.75	.40 [0.11, 0.67]	.55 [0.33, 0.89]	.05

*Note*. *h*^2^_g_ - group heritability; *c*^2^_g_ - group environmentality; *e*^2^_g_ - group nonshared environmentality.

**Table 7:** Univariate DeFries-Fulker extremes analysis: LI at 12 as a whole, and comparing persistent and late-onset LI.

Stability	*N*		Transformed co-twin mean		*h*^2^_g_ (95% CI)	*c*^2^_g_ (95% CI)	*e* ^2^_g_
	MZ	DZ	MZ	DZ			
LI at 12 years	239	219	.68	.50	.35 [0.28, 0.45]	.33 [0.25, 0.42]	.32
Persistent LI	60	28	.75	.62	.25 [0.05, 0.56]	.50 [0.23, 0.77]	.25
Late LI	179	191	.65	.48	.34 [0.09, 0.43]	.31 [0.11, 0.48]	.35

## Footnotes

1 We summarized internal consistency and test-retest reliability statistics, as well as key information on validity, from the manuals in the description of the tests. We also include internal reliability coefficients for the web version of each test, using TEDS data. These are very similar to the published figures for the paper-and-pencil versions.

2 By definition, the psychometric LI group was not referred for professional involvement. We do not have information on why the parental concerns noted for this group were not followed up further (e.g., whether the problem was perceived to be mild).

3 This could not be done for the parent referral-only group because this group is based on a dichotomous variable rather than a continuous one, which is required for DF extremes analysis.

4 As noted earlier, the different analysis method was necessary because DF extremes analysis requires continuous data, whereas liability threshold modeling is based on categorical data: Our verbal ability measure was continuous, but the parent referral data were categorical.

5 This excluded a small group of children who met both psychometric and parent-referral criteria at 4 years and criteria for LI at 12 years. This was done to ensure consistency of definitions between the 4- and 12-year analyses.
